# Growing into the American dream: childhood socialization and the formation of Clyde Griffiths in *An American tragedy*

**DOI:** 10.3389/fsoc.2026.1982929

**Published:** 2026-10-23

**Authors:** Hulkar Salimova, Ulugbek Ochilov

**Affiliations:** 1 Department of Foreign Languages and Social Sciences, Department of Foreign Languages and Social Sciences, Asia International University, Bukhara, Uzbekistan; 2 Interfaculty Department of Foreign Languages, Bukhara State University, Bukhara, Uzbekistan

**Keywords:** American dream, aspiration, consumer culture, family socialization, social class, social comparison

## Abstract

Theodore Dreiser’s *An American Tragedy* (1925) is one of the best-known American novels dealing with social ambition. It follows Clyde Griffiths, the son of poor street preachers in Kansas City, as he transitions from a strict religious childhood into adulthood, ending with his trial and execution for the death of a young woman. Existing interpretations often overlook what needs the most explanation: how Clyde came to desire what the American Dream offers. This Perspective allows us to explore aspiration as a product of socialization rather than as a psychological fact. Book One is useful for the sociology of families because it follows Clyde across several social settings over time. His poor evangelical family shapes his first understanding of morality, religion, and social class; later, work, a luxury hotel, peers, and consumer life expose him to different ideas of success and belonging. The article therefore asks what happens when values and expectations learned in the family meet different standards of status and belonging in the outside world. The argument distinguishes between the desire for goods and the desire for the social identity they represent and suggests that Clyde’s subsequent behavior is only comprehensible after this developmental process has taken place. On this account, achievement ideologies are not just adopted or rejected by individuals but are learned over time, in particular settings where certain lives are made “visible” and “desirable.” Fiction does not provide population-level evidence, but it can show how family socialization interacts with work, peers, and consumption over time.

## 1 Introduction

Theodore Dreiser (1871–1945) was a leading American novelist of the early twentieth century, and *An American Tragedy* (Dreiser, 1925) is widely regarded as one of his most important works. Based in part on a real murder case from 1906, the novel tells the story of Clyde Griffiths from boyhood to his execution. Clyde is the son of Asa and Elvira Griffiths, poor evangelical preachers who run a small street mission in Kansas City and take their children with them to sing and pray in public. Book One follows his teenage years in the city: He leaves school early, works in a drugstore then as a bellboy in a luxury hotel, and comes to admire the clothes, money, and confidence of the people around him. In Books Two and Three, he moves to Lycurgus, New York, to work in his rich uncle’s collar factory, becomes involved with two young women from very different social positions, and is finally tried and executed for the drowning of one of them.

The novel is worth reading closely for the sociology of families because it shows a young person moving through different social settings as he grows up. Clyde’s family shapes his earliest understanding of morality, religion, class, and social life. Later, his jobs, the hotel, his friends, and new patterns of consumption expose him to other ideas of success and belonging. The novel therefore raises a clear question for family sociology: what happens when the values, identities, and expectations learned in the family meet competing standards of status, consumption, success, and belonging in the outside world? In this reading, Clyde’s family is not simply background information. It is his first socializing environment, giving him a moral vocabulary and a sense of social position through which his later experiences become meaningful. This follows work that treats primary socialization as the child’s first world (Berger and Luckmann, 1966) and the family as a cultural institution through which values are passed from one generation to the next (Salimova and Ochilov, 2026). A novel cannot show how families shape aspiration in real populations. Dreiser’s narrative shows this process as it develops over time: The reader can follow, step by step, how the lessons Clyde learned at home interacts with his experiences in the workplace, his relationships with peers, and the world of consumption as he grows up.

Failure is a comparative judgment rather than a moral one. The suggestion that Clyde Griffiths was a young man broken by the American Dream presumes that he wanted what the Dream offers and that his desire requires no explanation. However, Dreiser’s novel implies just the opposite. The 19 chapters of Book One focus on the years before Clyde’s arrival in Lycurgus, before there is a factory, a Sondra, or a lake. They are not merely a prelude but the place where aspiration takes shape.

Dreiser scholarship has primarily examined his fiction through naturalism, with the ideology of desire and *Sister Carrie* as the main focus (Zeng, 2021; Zhao and Zhang, 2021; McNeilly, 2022). Studies on American fiction and achievement ideology have demonstrated that novels embody mobility myths both when the ideology is asserted and when it is lived (Cain, 2020; Roldán-Sevillano, 2025). In turn, sociologists have charted the childhood origins of mobility and later success (Chetty et al., 2018), the ritualization of the meritocratic belief (Martín de la Rosa and Lázaro, 2022), and the endurance of the Dream as cultural commitment (Wyatt-Nichol, 2011; Wolak and Peterson, 2020; Salimova and Ochilov, 2026). Less explicit attention has been paid to the process that links these bodies of work: the temporally cumulative process by which a young person first learns the standards that make one social identity seem desirable and another seem inadequate, learns to see his own life as falling short of the desired identity and learns to restructure his conduct around it. Readings of Clyde as a casualty of the Dream tend to take this desire as given; the present analysis addresses that gap.

This Perspective aims not so much to account for Clyde’s failure as to reconstruct the social production of the aspiring Clyde. The first question takes aspiration as the starting point, while the second treats it as something to be explained, as a sociological outcome rather than a given psychological preference.

This Perspective argues that Clyde cannot be said to have failed at the American Dream until he desires it, and that the first book of Dreiser’s novel traces this socialization in discernible phases rather than as a sudden conversion. The guiding question is accordingly: how does Dreiser represent the gradual social production of Clyde’s aspiration, and through which familial, occupational, peer, and consumer settings does the American Dream become a desirable social identity for him? Because the family is the first of these settings, the reading also shows how values learned at home give meaning to the competing standards Clyde meets later.

## 2 Analytical approach

The analysis is an interpretive, literary-sociological close reading of Book One of *An American Tragedy* (Dreiser, 1925), whose 19 chapters cover Clyde’s Kansas City years. Book One was chosen because the question is about the creation of aspiration, not its later effects in Lycurgus.

Book One was read chronologically, and passages were retained where the narrator records Clyde’s perception of social position, appearance, work, consumption, peers, family obligation, or belonging. These episodes were grouped, as an interpretive step rather than a formal coding procedure, around family and religious socialization and shame (Section 4.1), occupational exposure to status (4.2), peers as a reference group (4.3), consumption and identity performance (4.4), and moral conflict (Section 5). Each group is read against the concepts in Section 3, and their order is used to reconstruct a developmental sequence. The reading compares these episodes to those that challenge a socialization explanation: Clyde’s persistent moral sensibility, the money withheld from Esta, and the different reactions to the car accident (Section 5).

A novel does not provide population-level evidence. A narrative can follow a character over time and across places, making his interpretive world visible in ways that survey and interview data may capture less readily, and what is analytically useful about a narrative, precisely as representation.

## 3 Socialization into aspiration: theoretical framework

In primary socialization, the child is given the parents’ world as the world and only later encounters alternatives that reveal it was particular all along (Berger and Luckmann, 1966). Religious upbringing provides a particularly strong example, giving not only beliefs but also an evaluative vocabulary, and rejection of those beliefs does not negate its effect on subsequent moral development (Smith, 2023). In adolescence, peers are not simply imitated or rejected (Benish-Weisman, 2024), and identity in this period is better understood as a negotiation among the groups a young person regard themselves as belonging to, rather than as a fixed disposition (Crocetti et al., 2022).

This process is less a matter of calculation than of classed perception. Bourdieusian study shows how habitus conceals the objectivity of positions within the subjectivity of dispositions, making judgments about what is fine, vulgar, comfortable, or impossible seem immediate rather than reasoned (Fowler, 2020; Wacquant, 2018). Recent empirical research has highlighted this non-declarative aspect of cultural capital (Van der Waal et al., 2024; Ochilov et al., 2026a), and studies of youth view cultural capital in adolescence as actively produced rather than passively inherited (Prieur et al., 2023; Ochilov et al., 2026b). The central tension is that a young person can learn to value high-status ways of life long before they have the means to access them, and such recognition without access is a unique social situation, not simply a lack of resources (Bellino, 2020; Watkins, 2026; Ochilov, 2026).

The American Dream is here regarded as a cultural script, a socially transmitted story of the good life and how it is rightfully achieved. Families, ceremonies, schooling, and public discourse pass it on (Martín de la Rosa and Lázaro, 2022), and public trust in it does not necessarily follow economic conditions (Wolak and Peterson, 2020; Shanahan and Wilson, 2025). The desire for an object and the desire for the social identity an object signifies follow different logics (Eckhardt and Bardhi, 2020). Lamont (2019) has pointed out that the issue extends beyond possession to self-worth and recognition.

## 4 From the mission streets to the hotel: learning social difference

The textual findings appear in the order they appear in Book One; their interpretation is presented in Section 6.

### 4.1 Family, religion, and initial shame

Dreiser begins with a commercial street at dusk, and the boy does not look up, half-singing (Book One, ch. 1). Clyde is not first a material desiring subject but a person being looked at. Onlookers remark on the presence of the children; an earlier neighborhood has had children mock his father for his constant “Praise the Lord”. In the boy’s own summary, the work seems shabby to him, and it is degrading to be part of it.

This shame is socially situated and requires a standard; the novel provides one in the same place: the cars passing by, the people moving toward spaces of leisure, the “gay pairs of young people” laughing. Clyde is recording “something different, better, more beautiful” than his life. He does not yet desire the car itself but to be one of those who have it. The family’s poverty is exacerbated because the parents were “always making so much of religion and now at last they were making a business of it”. Private deprivation means one thing; publicly displayed deprivation carries the additional burden of exposure.

Material limits have become interpretive limits by Book One, ch. 2. Clyde discovers that his parents’ migration was motivated by religious rather than economic reasons, and he draws a conclusion about the labor market that is a concise instance of class socialization: he feels himself “above the type of labor which was purely manual” because boys like himself are clerks and druggists’ assistants. He has internalized this hierarchy, and the narrator attributes its origin to the general American attitude towards work.

### 4.2 Employment and exposure to status

The workplace then becomes the main site of socialization. The first place where he can observe such lives rather than merely glimpse them is the drugstore fountain (Book One, ch. 4). He observes matinee girls talking about parties and shows and decides that evening dress is “the last word in all true distinction”. He infers that without these outward signs of status, no pretty girl would want anything to do with him. For Clyde, consumption becomes a condition of social membership.

The Green-Davidson marks the next stage in this progression. Indoors, he is amazed by the marble, the orchestra, the well-dressed crowd (Book One, chs. 4–6). The result is a condensed image of social stratification: this “was what it meant to be rich” and “you did what you pleased” and “others like him waited on you”. In Veblenian terms, the hotel functions as an architecture of display that makes consumption legible to onlookers (Varul, 2006). Clyde works to maintain that display and learns from it.

### 4.3 Peers as a reference group

The decisive change at the hotel is Clyde’s reference group. Clyde’s peers are no longer the mission’s “down-and-out labourers, loafers, drunkards” but better-paid, socially confident bellboys. They teach Clyde the technicalities of their trade, such as how to get a tip and whom to pay off, as well as broader social lessons (Book One, chs. 5–7). Ratterer, Hegglund, and, most of all, Doyle become role models. Clyde dresses “as close as he could to the style of his mentor”, including the brown suit, cap, socks and stickpin. It is peer socialization based on taste, not opinion (Benish-Weisman, 2024), and also a modest accumulation of cultural capital unavailable within his family environment (Prieur et al., 2023).

The hotel also keeps Clyde in constant company of a class he cannot be a part of. He brings in the furs and the drinks, and looks a party of young people, not much older than himself, dancing in a room he is paid to enter and must leave. He compares himself to Doyle’s ease, the clothes of the guests and the young men in the tea-room who are making their way as men about town.

### 4.4 Consumption and status identity

His income rises rapidly and must be hidden. Tips soon outpace those of other members of the family, and he immediately downplays them so that his responsibilities to the family would not increase with his earnings (Book One, ch. 8). It protects his emerging aspirations from family claims, and it starts a trend of dealing with two audiences.

Clyde desires Hortense Briggs partly as a status marker, and she treats Clyde’s interest partly as a transaction, as her insistence that he buy the fur coat shows (Book One, chs. 14–16). In this episode, affection and social display become difficult to separate. Dreiser notes that Clyde knows she is coarse yet still pursues her. The desire for the status associated with having such a partner has replaced any judgment of the girl.

## 5 The moral problem of aspiration

In a chilly mission hall (ch. 16), Elvira Griffiths asks Clyde for 50 dollars for Esta, who is pregnant, abandoned, and needs a doctor. Clyde, who has promised Hortense the coat, has just 50 dollars in his pocket. He says he has none, gives her five, and writes to himself, “low, really mean”.

The obvious frame is Merton's (1938) “disjunction between culturally prescribed goals and legitimate means,” more recently interpreted as class-specific in content (Rushing, 1971; Murphy and Robinson, 2008). However, the material means are available. It is the identity that the money would enable him to perform that is blocked. In this case, the ‘strain’ is not between means and goals but between two identities: one based on family duty and one based on a new status identity. Dreiser also does not supply the self-justifying rationalizations that moral disengagement involves (Bandura, 1999; Tong et al., 2024).

It is that withholding which makes this reading non-deterministic. Here, dishonesty arises not simply from deprivation but from Clyde’s commitment to an emerging status identity, and Dreiser indicates that this commitment still involves choice. This is emphasized at the end of Book One when the stolen car tips over and a child is killed (ch. 19). The boys in the car share the same social world, yet they respond differently. Ratterer gets out and drags the others out. Hegglund turns around to help as he tries to get them to leave. Clyde assesses his own exposure, hesitates, and then runs. Shared socialization creates possibilities but does not make decisions. A deterministic explanation would suggest that boys with the same peer formation would respond in the same way, but this is not the case in the text. Structured agency is still agency, and moral responsibility within the fiction is not undermined by an explanation of how Clyde’s dilemmas became thinkable.

## 6 Discussion

Read against the framework in Section 3, each setting in Book One provides a new standard of comparison rather than simply a new object of desire. Cross-class encounters like those at the hotel teach about hierarchy and its barriers (Vassenden and Jonvik, 2018), and forms of closure sustain upper-class reproduction despite such proximity (Flemmen et al., 2017); Clyde’s classed perception is not a step toward membership but a stable position. Comparison, not absolute position, creates relative deprivation (Smith and Huo, 2014; Kim et al., 2018; Otten, 2020), and approaching a reference group without being part of it is a known source of status anxiety (Melita et al., 2023). Hidden tips are part of the same process, as family members not part of a relative’s upward mobility can also experience it as withdrawal (Born, 2023).

Recent research has documented how young people are constantly exposed to lifestyles they cannot afford and how visible consumption becomes a basis for social comparison and self-evaluation (Lamont, 2019; Bainotti, 2023; Gorga et al., 2024; Xu and Li, 2024). The same is true in Clyde’s Kansas City, where comparison technologies have changed, but repeated exposure and comparison operate through a similar process. The sociological problem remains the same: individuals build their self-concept in relation to socially organized fields of visibility involving reference groups to which they lack access.

## 7 Conclusion: socializing the American dream

Achievement ideologies are typically explored as objects of belief, whether they are endorsed and with what consequences. Book One of Dreiser’s novel offers an alternative: such ideologies are learned incrementally, in contexts that make certain lives visible, comparable, and desirable, and by the time a person is asked if he believes in the American Dream, much of the formative work has already occurred.

Clyde’s parents give him a moral vocabulary he cannot use: the street with shame, employment with exposure, peers with models, and money with a means of partial imitation. That sequence is not caused by aspiration but is the residue.

A single novel cannot empirically establish a social mechanism, and Clyde’s fictional trajectory does not establish how aspiration develops in real populations. It can, however, render a process visible in sufficient detail to describe, and this description is a necessary precondition for asking about the distribution of the process.

## Data Availability

The original contributions presented in the study are included in the article/supplementary material, further inquiries can be directed to the corresponding author.
